# Acute Complications in Patients with Myocardial Infarction with Non-Obstructive Coronary Arteries: A Systematic Review with Special Focus on Mechanical Complications

**DOI:** 10.31083/j.rcm2312393

**Published:** 2022-12-02

**Authors:** Jacek Bil, Patryk Buller, Robert J. Gil, Leszek Gromadziński, Dariusz Onichimowski, Rakesh Jalali, Adam Kern

**Affiliations:** ^1^Department of Invasive Cardiology, Centre of Postgraduate Medical Education, 02-507 Warsaw, Poland; ^2^Cardiology Clinic, Voivodeship Hospital, 09-400 Płock, Poland; ^3^Department of Cardiology and Internal Medicine, School of Medicine, Collegium Medicum, University of Warmia and Mazury, 11-082 Olsztyn, Poland; ^4^Department of Anaesthesiology and Intensive Care, School of Medicine, Collegium Medicum, University of Warmia and Mazury, 11-082 Olsztyn, Poland; ^5^Clinical Department of Anaesthesiology and Intensive Care, Regional Specialist Hospital, 10-651 Olsztyn, Poland; ^6^Emergency Medicine Department, School of Medicine, Collegium Medicum, University of Warmia and Mazury, 11-082 Olsztyn, Poland; ^7^Clinical Emergency Department, Regional Specialist Hospital, 10-561 Olsztyn, Poland; ^8^Department of Cardiology, Regional Specialist Hospital, 10-561 Olsztyn, Poland

**Keywords:** MINOCA, INOCA, acetylcholine, MI complications, microcirculation dysfunction

## Abstract

**Background:**

Recently, we have observed an increasing focus on myocardial infarction (MI) with non-obstructive coronary arteries (MINOCA) patients. MINOCA incidence is estimated to be within the range of 5–15% of all MI cases. Unfortunately, MINOCA relates to various conditions that are not rarely hard to identify, including coronary microcirculation dysfunction, epicardial coronary spasm, or plaque erosion. Our systematic review aimed to identify and appraise previous studies which characterized acute complications, with particular focus on mechanical complications, in patients with MINOCA.

**Methods:**

Applying the MeSH strategy in PubMed and Embase, two operators independently and systematically reviewed published studies on patients diagnosed with MINOCA and in whom acute complications were described. Papers published in the last 10 years (June 2012–June 2022) to reflect the introduction of the MINOCA definition as well as the current clinical practice were analyzed. The research was conducted in July 2022.

**Results:**

The search yielded 192 records. After abstract review, 79 papers were left, and after full-text analysis, we finally included 20 studies. Among 20 studies, there were: one randomized controlled trial, one prospective study, five retrospective studies, 1 case series, and 12 case reports with a total number of 337,385 patients. In the identified literature, we revealed 7 cases of intraventricular septal rupture, 3 cases of free wall rupture with pericardial effusion or cardiac tamponade, and 3 cases of bleeding complications (intracerebral or intestinal bleeding). Moreover, the ventricular arrhythmia incidence ranged from 2% to 13.8%, and the in-hospital death rate ranged from 0.9% to 6.4%.

**Conclusions:**

These findings suggest that MINOCA patients should be treated as standard MI patients with watchful monitoring, especially in the first few days.

## 1. Introduction

Myocardial infarction (MI) with non-obstructive coronary arteries (MINOCA), a heterogeneous syndrome evoked in several different pathophysiological pathways, is defined by clinical/laboratory evidence of MI and no significant coronary artery stenosis (lesions with diameter stenosis <50%) [1].

In recent years, we have observed an increasing focus on MINOCA patients. MINOCA incidence is estimated to be within the range of 5–15% of all MI cases [2]. Unfortunately, MINOCA relates to various conditions that are not rarely hard to identify, including coronary microcirculation dysfunction, epicardial coronary spasm, or plaque erosion [3]. Recently, the paper on management of patients with ischemia and non-obstructive coronary arteries was published [4].

The MINOCA perception evolved from a benign disorder, and cardiologists acknowledged that MINOCA patients characterize similar long-term outcomes as patients with obstructive coronary artery disease (CAD). In a recent Italian study, cardiac death rate was lower in the MINOCA group (4.2% vs. 8.4%, *p* = 0.03); however, there were no significant between other endpoints, such as: recurrent MI (17.3% vs. 25.4%, *p* = 0.18), ischemic stroke (9.5% vs. 3.7%, *p* = 0.12) or all-cause death rate (14.1% vs. 20.7%, *p* = 0.26) with a median follow-up of 19.9 years [5]. Also, Safdar *et al*. [6] showed that 1-month (1.1% vs. 1.7%, *p* = 0.43) and 12-month (0.6% vs. 2.3%) mortality rates were similar between MINOCA and obstructive MI groups. The other studies also confirmed these findings [7, 8].

The other question concerns early outcomes and acute complications typical for complete coronary artery occlusion and myocardial ischemia, e.g., wall rupture or acute mitral regurgitation. Our systematic review aimed to identify and appraise previous studies which characterized acute complications, with particular focus on mechanical complications, in patients with MINOCA.

## 2. Materials and Methods

We performed a systematic review according to the preferred reporting items for systematic reviews and meta-analysis guidelines (PRISMA) [9]. Applying the MeSH strategy in PubMed and Embase, two operators (J.B. and A.K.) independently and systematically reviewed published studies on patients diagnosed with MINOCA and in whom acute complications were described. The terms searched were ((complications) OR (((((((perforation) OR (rupture)) OR (arrhythmia)) OR (tamponade)) OR (pericarditis)) OR (aneurysm)) OR (mitral regurgitation))) AND ((((myocardial infarction with nonobstructive coronary arteries)) OR (MINOCA) OR (myocardial infarction) OR (MI) AND (non-obstructive coronary artery) OR (non-obstructive coronary arteries)))) as well as (MINOCA AND registry). We analyzed papers published in the last 10 years (June 2012–June 2022) to reflect the introduction of the MINOCA definition as well as the current clinical practice. The research was conducted in July 2022. The detailed search results are presented in **Supplementary Table 1**.

The inclusion criteria were (1) studies including patients with MINOCA and (2) studies with acute MI complications. The exclusion criteria were (1) meta-analyses, (2) reviews or editorials, (3) the sample population duplication, (4) conference abstracts, (5) animal studies, (6) duplicates, and (7) grey literature. Only papers published in English and peer-reviewed journals were retrieved.

Two operators completed a database with the data regarding the authors, study type, publication year, number of patients, complications, and MINOCA exact cause if available. The primary purpose of this systematic review was to describe acute complications, with a particular focus on mechanical complications, in patients with MINOCA.

Clinical data were expressed numerically, including patient characteristics, procedural features, and complications. Categorical variables are shown as percentages, and continuous variables-as means.

## 3. Results

### 3.1 Search Strategy Results

The search yielded 192 records. After abstract review, 79 papers were left, and after full-text analysis, we finally included 20 studies. The quality of the included studies was verified using MINORS criteria, with overall scores ranging between 10 and 18 (**Supplementary Table 2**) [10].

### 3.2 Included Study Details

Among 20 studies [7, 11, 12, 13, 14, 15, 16, 17, 18, 19, 20, 21, 22, 23, 24, 25, 26, 27, 28, 29], there were: one randomized controlled trial (RCT) [12], one prospective study [15], five retrospective studies [7, 11, 13, 14, 16], 1 case series [21], and 12 case reports [17, 18, 19, 20, 22, 23, 24, 25, 26, 27, 28, 29] with a total number of 337,385 patients. The results of observational studies are presented in Table 1 (Ref. [7, 11, 12, 13, 14, 15, 16]), and the results of case reports—in Table 2 (Ref. [17, 18, 19, 20, 21, 22, 23, 24, 25, 26, 27, 28, 29]).

**Table 1.** **Acute complications in patients with MINOCA—observational studies**.

No	Authors	Year	Study type	No of patients	Complications (timing)		MINOCA Additional information
1.	Li *et al*. [7]	2022	Retrospective study	107	In-hospital:		73 patients (68.2%)—NSTE-MINOCA
						Cardiac death (0.9%)	34 patients (31.8%)—STE-MINOCA
						Malignant arrhythmia (2.8%)	
						Heart failure (1.9%)	
2.	Jędrychowska *et al*. [11]	2021	Retrospective study	130,358	In-hospital:		MINOCA incidence—4.4%
						Cardiac arrest (0.2%)	MI-CAD cardiac arrest—0.8%
						Death (0.2%)	MI-CAD death—1.3%
3.	Bossard *et al*. [12]	2021	RCT	25,382	30-days observation:		MINOCA incidence—6.7%
						All-cause death (0.6%)	STE-MINOCA—8.8% of MINOCA cases
						Cardiac death (0.6%)	
						MI (0.5%)	
						Stroke (0.4%)	
						Recurrent ischemia (0.3%)	
4.	Gasior *et al*. [13]	2020	Retrospective study	166,949	In-hospital:		MINOCA incidence—2.94%
						Cardiac arrest (0.90%)	STE-MINOCA—16.55% of MINOCA cases
						Pulmonary edema (0.25%)	
						Cardiogenic shock (0.36%)	
						MI (0.06%)	
						Death (1.67%)	
						Cardiac death (1.99%)	
						Stroke (0.21%)	
5.	Ishii *et al*. [14]	2020	Retrospective study	14,045	In-hospital death (6.4%)		MINOCA incidence—10.2%
						Death within 24 h: 1.75/1000 person-days	In-hospital death in MI-CAD—6.2%
6.	Choo *et al*. [15]	2019	Prospective study	396	In-hospital:		MI-CAD:
						Cardiogenic shock (5.1%)	Cardiogenic shock—8.9%
						New-onset heart failure (3.5%)	In-hospital death —3.5%
						Cerebral infarction (1%)	
						Cerebral hemorrhage (0.3%)	
						Ventricular arrhythmia (2%)	
						Death (2.8%)	
7.	Bière *et al*. [16]	2017	Retrospective study	131	In-hospital:		34 (25.9%)—MI
						Pericardial effusion (15.3%);	47 (35.9%)—myocarditis
						Ventricular arrhythmia (13.8%; 17—ventricular tachycardia, 1—ventricular fibrillation)	50 (38.2%)—“no LGE” group

NSTE-MINOCA, non-ST-elevation myocardial infarction with non-obstructive coronary arteries; RCT, randomized controlled trial; STE-MINOCA, ST-elevation myocardial infarction with non-obstructive coronary arteries; MI-CAD, myocardial infarction with obstructive coronary artery disease; LGE, late gadolinium enhancement.

**Table 2.** **Acute complications in patients with MINOCA—case reports**.

No	Authors	Year	Study type	No of patients	Complications	Timing	MINOCA cause/comments
1.	Kafkas *et al*. [17]	2022	Case report	1	Intraventricular septal rupture	(30 h from initial symptoms)	30 h after admission; surgical treatment 5 days later
2.	Petrov *et al*. [18]	2021	Case report	1	Intraventricular septal rupture	(48 h from symptoms)	Probable coronary embolization in the course of AF; endovascular repair of ventricular septal defect
3.	Giavarini *et al*. [19]	2021	Case report	1	Free wall rupture	(24 h from admission)	ICD implantation in 2012 due to ventricular fibrillation ascribed to vasospastic angina
4.	Codecasa *et al*. [20]	2021	Case report	1	Intraventricular septal rupture	(2 days from symptoms)	48 h after admission
5.	Aimo *et al*. [21]	2020	Case series	5	4 sudden cardiac death related likely to ventricular arrhythmias; 1 free wall rupture	(2 days from symptoms)	All linked with strong emotional stress; death took place 72 h after admission
6.	Piels *et al*. [22]	2019	Case report	1	Ventricular arrhythmia causing sudden cardiac arrest	(5 days from initial presentation)	Probably plaque erosion/dissection in the left main stem; MI confirmed in an autopsy
7.	Ozdemir *et al*. [23]	2019	Case report	1	Ventricular fibrillation	(4–5 h)	Vasospastic angina, ST-elevation
8.	Li *et al*. [24]	2019	Case report	1	Ventricular tachycardia	(1–2 weeks from initial admission)	Complicated by heart failure, ventricular aneurysm, and ventricular thrombus
9.	Roth *et al*. [25]	2018	Case report	1	Free wall rupture with cardiac tamponade and subsequent death	(<24 h from symptoms)	Rupture diagnosed at admission
10.	Kalvin *et al*. [26]	2017	Case report	1	Intraventricular septal rupture	(12 h after admission)	12 h after admission, developed mild hypotension and holosystolic murmur
11.	Rodríguez *et al*. [27]	2016	Case report	1	Intraventricular septal rupture	(4 h from symptoms)	Ventriculography disclosed ventricular septal defect
12.	Viveiros *et al*. [28]	2015	Case report	1	Large LV apical aneurysm (with thrombus) and ventricular septal defect with 2 jets to apical RV pseudoaneurysm (with thrombus)	(<30 days from symptoms)	Corrective surgery: Dor procedure (endoventricular circular patch plasty) joined with ventricular septal defect closure and resection of the RV pseudo-aneurysm
13.	Akilli *et al*. [29]	2013	Case report	1	Intraventricular septal rupture	(2 days from initial symptoms)	Probable coronary embolization during AF

NSTE-MINOCA, non-ST-elevation myocardial infarction with non-obstructive coronary arteries; STE-MINOCA, ST-elevation myocardial infarction with non-obstructive coronary arteries; ICD, implantable cardioverter defibrillator; MI-CAD, myocardial infarction with obstructive coronary artery disease; LV, left ventricle; RV, right ventricle; AF, atrial fibrillation.

In the literature search, we also identified papers showing complications not associated directly with MINOCA pathomechanism, but rather with MINOCA management. Jung *et al*. [30] reported intracerebral hemorrhage, and Kissami *et al*. [31] reported intestinal bleeding. Both could have been related to dual antiplatelet therapy and heparin treatment.

## 4. Discussion

Among 20 studies, there were: one RCT, one prospective study, five retrospective studies, 1 case series, and 12 case reports with a total number of 337,385 patients. The identified literature revealed 5 cases of intraventricular septal rupture, 3 cases of free wall rupture with pericardial effusion or cardiac tamponade, and 3 cases of bleeding complications (intracerebral or intestinal bleeding). Moreover, the ventricular arrhythmia incidence ranged from 2% to 13.8%, and the in-hospital death rate ranged from 0.9% to 6.4%.

Patients with MI should be monitored in the first days after admission, mainly due to possibly atrioventricular conduction abnormalities and ventricular arrhythmias. The recent European Society of Cardiology guidelines on non-ST-elevation acute coronary syndromes (NSTE-ACS) recommend rhythm monitoring up to 24 h in patients at low risk for cardiac arrhythmias, and >24 h in patients at increased risk [32]. Gathered publications show that this is also the case for MINOCA patients. Bière *et al*. [16] observed that 13.8% of MINOCA patients developed ventricular arrhythmia during the index hospitalization. Most frequently, there were cases of ventricular tachycardia, but one case of ventricular fibrillation was also registered. Most arrhythmias occurred during the first days following admission to the hospital. This research also proved that, when left ventricular ejection fraction is within normal limits, patients with MI and patients with myocarditis characterize similar arrhythmic risks. Following the initial phase of the episode, when the risk of ventricular arrhythmia was evident, the arrhythmic risk seemed very low during further hospitalization. Moreover, the authors demonstrated that ST-elevation was an independent risk factor of ventricular arrhythmias at early-stage disease with an excellent negative predictive value of 92% for sustained ventricular tachycardia. Additionally, in magnetic resonance imaging, it was proved that transmural late gadolinium enhancement (LGE) extent was an independent risk factor for ventricular arrhythmia during the acute period. These findings can be very helpful, if available soon after admission, in selecting at-risk patients requiring watchful and probably extended monitoring. In those patients, proper pharmacotherapy is crucial; however, the optimal strategy is still debatable. In that study population, 69.5% of subjects were administered β-blockers, taking into consideration the management of patients with recent MI. Taking into consideration a high rate of patients developing ventricular arrhythmia, such proceedings probably should be encouraged [16].

Interestingly, Li *et al*. [7] compared two groups of patients with MINOCA, i.e., with ST-elevation (STE-MINOCA) and non-ST-elevation (NSTE-MINOCA). In NSTE-MINOCA patients, one could have observed a trend toward a worse prognosis in the long-term follow-up. The malignant arrhythmia rate in NSTE-MINOCA patients was 4.8%, whereas, in STE-MINOCA patients, it was 0%. Here, STE-MINOCA patients were younger, characterized by lower N-terminal pro-brain natriuretic peptide (NT-proBNP) and smaller left atrial diameter. Also, STE-MINOCA patients more frequently received dual antiplatelet therapy.

Apart from ventricular arrhythmias, myocardial rupture (intraventricular septal rupture or free wall rupture) is a characteristic MI complication, especially when the infarcted area is large. Simultaneously, intraventricular septal rupture is one of the most serious complications in the MI course. The reported mortality substantially improved in the last two decades, mainly due to wide access to cath-labs and percutaneous revascularization in the acute setting. Mortality rates ranged from 1% to 3% in the pre-reperfusion times, and now has decreased to 0.17%–0.31% [33]. Probably the first case of such complication in a MINOCA patient was described in 1984 [34]. In our literature search, we identified 7 such cases [17, 18, 20, 26, 27, 28, 29]. Moreover, we identified 3 cases of free wall rupture with accompanied pericardial effusion or cardiac tamponade [19, 21, 25]. Most cases were obsereved during first 48–72 h after initial symptoms [17, 18, 19, 20, 21, 25, 26, 27, 29]; however, certain complications appeared later [22, 24, 28]. This shows that also postdischarge follow-up is very important.

Potential pathomechanisms leading to intraventricular septal or free wall rupture in MINOCA patients might be either coronary artery spasm or plaque erosion (more frequently observed in NSTE-MINOCA, younger patients and without the presence of classical cardiovascular risk factors) or atrial fibrillation that evokes transient epicardial thrombosis and/or occlusion, including an ostial and isolated occlusion of a sizeable septal perforator not visualized on coronary angiography. Also, this can be caused be ostial occlusion of the coronary artery and might by unnoticed during coronary angiography. In MINOCA patients, the sudden onset of ischemia is often accompanied by following abrupt reperfusion. This might be linked with enhanced neutrophil activity in the ischemic area resulting in a surge of lytic enzymes and myocardial cell apoptosis. As the recent literature data show, ventricular septal defect in the course of MINOCA can also be repaired non only by open heart surgery but also with endovascular methods [18].

We also identified that bleeding complications linked to dual antiplatelet therapy or heparin treatment is also a problem in MINOCA patients. There are cases showing gastrointestinal as well as intracerebral hemorrhage events [30, 31].

Finally, it must be restated that MINOCA outcomes are no better than MI-CAD. Ishii *et al*. [14] showed that 894 patients with a working diagnosis of MINOCA (6.4%) and 7644 MI-CAD (6.2%) died in the course of the index hospitalization. In the same study, the authors disclosed that coronary spam and takotsubo cardiomyopathy were significant negative predictors of in-hospital mortality in patients with a working diagnosis of MINOCA, and myocarditis and aortic dissection were significantly positive predictors. Moreover, Xu *et al*. [35] showed that non-ST-elevation cases were more frequently observed than ST-elevation cases in the MINOCA population. In the 12-month follow-up, the authors observed no differences in the outcomes between the ST-elevation and non-ST-elevation MINOCA patients, with no significant differences in death rates and similar rates of major adverse cardiovascular events (20.9% vs. 19.3%, *p* = 0.77).

## 5. Conclusions

Acute complications, including mechanical complications, in MINOCA patients, are not casuistic. These findings suggest that MINOCA patients should be managed as standard MI patients with watchful monitoring, especially in the first few days.

## Abbreviations

AF, atrial fibrillation; CAD, coronary artery disease; ICD, implantable cardioverter defibrillator; INOCA, ischemia with non-obstructive coronary arteries; LGE, late gadolinium enhancement; LV, left ventricle; MI, myocardial infarction; MI-CAD, myocardial infarction with obstructive coronary artery disease; MINOCA, myocardial infarction with non-obstructive coronary arteries; NSTE-ACS, non-ST-elevation acute coronary syndromes; NSTE-MINOCA, non-ST-elevation myocardial infarction with non-obstructive coronary arteries; NT-proBNP, N-terminal pro-brain natriuretic peptide; RCT, randomized controlled trial; RV, right ventricle; STE-MINOCA, ST-elevation myocardial infarction with non-obstructive coronary arteries.

## Supplementary Material

Supplementary material associated with this article can be found, in the online version, at https://doi.org/10.31083/j.rcm2312393.
