# Supplementary material for: Acute Complications in Patients with Myocardial Infarction with Non-Obstructive Coronary Arteries: A Systematic Review with Special Focus on Mechanical Complications
Source: Rev Cardiovasc Med. 2022 Dec 2;23(12):393. doi: 10.31083/j.rcm2312393 (PMC11270480; doi:10.31083/j.rcm2312393)
Supplement: Supplementary file 1 [file 2153-8174-23-12-393-s1.zip › 2153-8174-23-12-393-s1/Supplementary Material.docx]

Supplementary Table 1. Detailed search results from PubMed database.

| **Search number** | **Query** | **Sort By** | **Filters** | **Search Details** | **Results** |
| --- | --- | --- | --- | --- | --- |
| **12** | MINOCA registry | Most Recent | in the last 10 years | (("minoca"[MeSH Terms] OR "minoca"[All Fields]) AND ("registries"[MeSH Terms] OR "registries"[All Fields] OR "registry"[All Fields] OR "registry s"[All Fields])) AND (y_10[Filter]) | 53 |
| **11** | (((((((((perforation) OR (rupture)) OR (arrhythmia)) OR (tamponade)) OR (pericarditis)) OR (aneurysm)) OR ("mitral regurgitation")) OR (complications)) OR ("Pericardial Effusion"[Mesh])) AND (((("myocardial infarction") OR (MI)) AND (("non-obstructive coronary artery") OR ("non-obstructive coronary arteries"))) OR ((("myocardial infarction with non-obstructive coronary arteries") OR ("myocardial infarction with non obstructive coronary arteries")) OR (MINOCA))) | Most Recent | in the last 10 years | (("perforant"[All Fields] OR "perforants"[All Fields] OR "perforate"[All Fields] OR "perforated"[All Fields] OR "perforates"[All Fields] OR "perforating"[All Fields] OR "perforation"[All Fields] OR "perforations"[All Fields] OR "perforative"[All Fields] OR "perforator"[All Fields] OR "perforator s"[All Fields] OR "perforators"[All Fields] OR ("ruptur"[All Fields] OR "rupture"[MeSH Terms] OR "rupture"[All Fields] OR "ruptured"[All Fields] OR "ruptures"[All Fields] OR "rupturing"[All Fields]) OR ("arrhythmia s"[All Fields] OR "arrhythmias, cardiac"[MeSH Terms] OR ("arrhythmias"[All Fields] AND "cardiac"[All Fields]) OR "cardiac arrhythmias"[All Fields] OR "arrhythmia"[All Fields] OR "arrhythmias"[All Fields]) OR ("tamponade"[All Fields] OR "tamponaded"[All Fields] OR "tamponades"[All Fields] OR "tamponading"[All Fields]) OR ("pericardic"[All Fields] OR "pericarditis"[MeSH Terms] OR "pericarditis"[All Fields]) OR ("aneurysm"[MeSH Terms] OR "aneurysm"[All Fields] OR "aneurysms"[All Fields] OR "aneurysm s"[All Fields] OR "aneurysmal"[All Fields] OR "aneurysmally"[All Fields] OR "aneurysmic"[All Fields]) OR "mitral regurgitation"[All Fields] OR ("complicances"[All Fields] OR "complicate"[All Fields] OR "complicated"[All Fields] OR "complicates"[All Fields] OR "complicating"[All Fields] OR "complication"[All Fields] OR "complication s"[All Fields] OR "complications"[MeSH Subheading] OR "complications"[All Fields]) OR "Pericardial Effusion"[MeSH Terms]) AND ((("myocardial infarction"[All Fields] OR ("microbiology"[MeSH Subheading] OR "microbiology"[All Fields] OR "mi"[All Fields])) AND ("non-obstructive coronary artery"[All Fields] OR "non-obstructive coronary arteries"[All Fields])) OR ("myocardial infarction with non-obstructive coronary arteries"[All Fields] OR "myocardial infarction with non-obstructive coronary arteries"[All Fields] OR ("minoca"[MeSH Terms] OR "minoca"[All Fields])))) AND (y_10[Filter]) | 139 |
| **10** | (((((((((perforation) OR (rupture)) OR (arrhythmia)) OR (tamponade)) OR (pericarditis)) OR (aneurysm)) OR ("mitral regurgitation")) OR (complications)) OR ("Pericardial Effusion"[Mesh])) AND (((("myocardial infarction") OR (MI)) AND (("non-obstructive coronary artery") OR ("non-obstructive coronary arteries"))) OR ((("myocardial infarction with non-obstructive coronary arteries") OR ("myocardial infarction with non obstructive coronary arteries")) OR (MINOCA))) |  |  | ("perforant"[All Fields] OR "perforants"[All Fields] OR "perforate"[All Fields] OR "perforated"[All Fields] OR "perforates"[All Fields] OR "perforating"[All Fields] OR "perforation"[All Fields] OR "perforations"[All Fields] OR "perforative"[All Fields] OR "perforator"[All Fields] OR "perforator s"[All Fields] OR "perforators"[All Fields] OR ("ruptur"[All Fields] OR "rupture"[MeSH Terms] OR "rupture"[All Fields] OR "ruptured"[All Fields] OR "ruptures"[All Fields] OR "rupturing"[All Fields]) OR ("arrhythmia s"[All Fields] OR "arrhythmias, cardiac"[MeSH Terms] OR ("arrhythmias"[All Fields] AND "cardiac"[All Fields]) OR "cardiac arrhythmias"[All Fields] OR "arrhythmia"[All Fields] OR "arrhythmias"[All Fields]) OR ("tamponade"[All Fields] OR "tamponaded"[All Fields] OR "tamponades"[All Fields] OR "tamponading"[All Fields]) OR ("pericardic"[All Fields] OR "pericarditis"[MeSH Terms] OR "pericarditis"[All Fields]) OR ("aneurysm"[MeSH Terms] OR "aneurysm"[All Fields] OR "aneurysms"[All Fields] OR "aneurysm s"[All Fields] OR "aneurysmal"[All Fields] OR "aneurysmally"[All Fields] OR "aneurysmic"[All Fields]) OR "mitral regurgitation"[All Fields] OR ("complicances"[All Fields] OR "complicate"[All Fields] OR "complicated"[All Fields] OR "complicates"[All Fields] OR "complicating"[All Fields] OR "complication"[All Fields] OR "complication s"[All Fields] OR "complications"[MeSH Subheading] OR "complications"[All Fields]) OR "Pericardial Effusion"[MeSH Terms]) AND ((("myocardial infarction"[All Fields] OR ("microbiology"[MeSH Subheading] OR "microbiology"[All Fields] OR "mi"[All Fields])) AND ("non-obstructive coronary artery"[All Fields] OR "non-obstructive coronary arteries"[All Fields])) OR ("myocardial infarction with non-obstructive coronary arteries"[All Fields] OR "myocardial infarction with non-obstructive coronary arteries"[All Fields] OR ("minoca"[MeSH Terms] OR "minoca"[All Fields]))) | 145 |
| **9** | ((((((((perforation) OR (rupture)) OR (arrhythmia)) OR (tamponade)) OR (pericarditis)) OR (aneurysm)) OR ("mitral regurgitation")) OR (complications)) OR ("Pericardial Effusion"[Mesh]) |  |  | "perforant"[All Fields] OR "perforants"[All Fields] OR "perforate"[All Fields] OR "perforated"[All Fields] OR "perforates"[All Fields] OR "perforating"[All Fields] OR "perforation"[All Fields] OR "perforations"[All Fields] OR "perforative"[All Fields] OR "perforator"[All Fields] OR "perforator s"[All Fields] OR "perforators"[All Fields] OR ("ruptur"[All Fields] OR "rupture"[MeSH Terms] OR "rupture"[All Fields] OR "ruptured"[All Fields] OR "ruptures"[All Fields] OR "rupturing"[All Fields]) OR ("arrhythmia s"[All Fields] OR "arrhythmias, cardiac"[MeSH Terms] OR ("arrhythmias"[All Fields] AND "cardiac"[All Fields]) OR "cardiac arrhythmias"[All Fields] OR "arrhythmia"[All Fields] OR "arrhythmias"[All Fields]) OR ("tamponade"[All Fields] OR "tamponaded"[All Fields] OR "tamponades"[All Fields] OR "tamponading"[All Fields]) OR ("pericardic"[All Fields] OR "pericarditis"[MeSH Terms] OR "pericarditis"[All Fields]) OR ("aneurysm"[MeSH Terms] OR "aneurysm"[All Fields] OR "aneurysms"[All Fields] OR "aneurysm s"[All Fields] OR "aneurysmal"[All Fields] OR "aneurysmally"[All Fields] OR "aneurysmic"[All Fields]) OR "mitral regurgitation"[All Fields] OR ("complicances"[All Fields] OR "complicate"[All Fields] OR "complicated"[All Fields] OR "complicates"[All Fields] OR "complicating"[All Fields] OR "complication"[All Fields] OR "complication s"[All Fields] OR "complications"[MeSH Subheading] OR "complications"[All Fields]) OR "Pericardial Effusion"[MeSH Terms] | 4,116,153 |
| **8** | ((((((perforation) OR (rupture)) OR (arrhythmia)) OR (tamponade)) OR (pericarditis)) OR (aneurysm)) OR ("mitral regurgitation") |  |  | "perforant"[All Fields] OR "perforants"[All Fields] OR "perforate"[All Fields] OR "perforated"[All Fields] OR "perforates"[All Fields] OR "perforating"[All Fields] OR "perforation"[All Fields] OR "perforations"[All Fields] OR "perforative"[All Fields] OR "perforator"[All Fields] OR "perforator s"[All Fields] OR "perforators"[All Fields] OR ("ruptur"[All Fields] OR "rupture"[MeSH Terms] OR "rupture"[All Fields] OR "ruptured"[All Fields] OR "ruptures"[All Fields] OR "rupturing"[All Fields]) OR ("arrhythmia s"[All Fields] OR "arrhythmias, cardiac"[MeSH Terms] OR ("arrhythmias"[All Fields] AND "cardiac"[All Fields]) OR "cardiac arrhythmias"[All Fields] OR "arrhythmia"[All Fields] OR "arrhythmias"[All Fields]) OR ("tamponade"[All Fields] OR "tamponaded"[All Fields] OR "tamponades"[All Fields] OR "tamponading"[All Fields]) OR ("pericardic"[All Fields] OR "pericarditis"[MeSH Terms] OR "pericarditis"[All Fields]) OR ("aneurysm"[MeSH Terms] OR "aneurysm"[All Fields] OR "aneurysms"[All Fields] OR "aneurysm s"[All Fields] OR "aneurysmal"[All Fields] OR "aneurysmally"[All Fields] OR "aneurysmic"[All Fields]) OR "mitral regurgitation"[All Fields] | 729,217 |
| **7** | complications |  |  | "complicances"[All Fields] OR "complicate"[All Fields] OR "complicated"[All Fields] OR "complicates"[All Fields] OR "complicating"[All Fields] OR "complication"[All Fields] OR "complication s"[All Fields] OR "complications"[MeSH Subheading] OR "complications"[All Fields] | 3,681,329 |
| **6** | ((("myocardial infarction") OR (MI)) AND (("non-obstructive coronary artery") OR ("non-obstructive coronary arteries"))) OR ((("myocardial infarction with non-obstructive coronary arteries") OR ("myocardial infarction with non obstructive coronary arteries")) OR (MINOCA)) |  |  | (("myocardial infarction"[All Fields] OR ("microbiology"[MeSH Subheading] OR "microbiology"[All Fields] OR "mi"[All Fields])) AND ("non-obstructive coronary artery"[All Fields] OR "non-obstructive coronary arteries"[All Fields])) OR ("myocardial infarction with non-obstructive coronary arteries"[All Fields] OR "myocardial infarction with non-obstructive coronary arteries"[All Fields] OR ("minoca"[MeSH Terms] OR "minoca"[All Fields])) | 515 |
| **5** | (("myocardial infarction") OR (MI)) AND (("non-obstructive coronary artery") OR ("non-obstructive coronary arteries")) |  |  | ("myocardial infarction"[All Fields] OR ("microbiology"[MeSH Subheading] OR "microbiology"[All Fields] OR "mi"[All Fields])) AND ("non-obstructive coronary artery"[All Fields] OR "non-obstructive coronary arteries"[All Fields]) | 290 |
| **4** | ("non-obstructive coronary artery") OR ("non-obstructive coronary arteries") |  |  | "non-obstructive coronary artery"[All Fields] OR "non-obstructive coronary arteries"[All Fields] | 498 |
| **3** | ("myocardial infarction") OR (MI) |  |  | "myocardial infarction"[All Fields] OR "microbiology"[MeSH Subheading] OR "microbiology"[All Fields] OR "mi"[All Fields] | 2,084,553 |
| **2** | (("myocardial infarction with non-obstructive coronary arteries") OR ("myocardial infarction with non obstructive coronary arteries")) OR (MINOCA) |  |  | "myocardial infarction with non-obstructive coronary arteries"[All Fields] OR "myocardial infarction with non-obstructive coronary arteries"[All Fields] OR "minoca"[MeSH Terms] OR "minoca"[All Fields] | 431 |
| **1** | "Pericardial Effusion"[Mesh] | Most Recent |  | "Pericardial Effusion"[MeSH Terms] | 9,127 |

Supplementary Table 2. Study quality assessment using MINORS criteria.

In every item the following points were assigned:

0 point – “Not reported”,

1 point – “Reported but inadequate”

2 points – “Reported and adequate”

Questions/Items:

1. A clearly stated aim;

2. Inclusion of consecutive patients;

3. Prospective collection of data;

4. Endpoints appropriate to the study aim;

5. Unbiased assessment of study endpoints;

6. Follow-up appropriate to the study aim;

7. Loss to follow up less than 5%;

8. Prospective calculation of the study size;

9. Adequate statistical analysis.

| **Reference** | | | | **Question/Item** | | | | | | | | | |
| --- | --- | --- | --- | --- | --- | --- | --- | --- | --- | --- | --- | --- | --- |
| **No** | **Authors** | **Year** | **Study type** | **1** | **2** | **3** | **4** | **5** | **6** | **7** | **8** | **9** | **Total** |
| 1. | Li M, *et al*. [5] | 2022 | retrospective study | 2 | 2 | 0 | 2 | 2 | 2 | 2 | 0 | 2 | 14 |
| 2. | Jung *et al*. [9] | 2022 | case report | 2 | 0 | 0 | 2 | 2 | 2 | 2 | 0 | 0 | 10 |
| 3. | Kafkas *et al*. [10] | 2022 | case report | 2 | 0 | 0 | 2 | 2 | 2 | 2 | 0 | 0 | 10 |
| 4. | Petrov *et al*. [11] | 2021 | case report | 2 | 0 | 0 | 2 | 2 | 2 | 2 | 0 | 0 | 10 |
| 5. | Giavarini *et al*. [12] | 2021 | case report | 2 | 0 | 0 | 2 | 2 | 2 | 2 | 0 | 0 | 10 |
| 6. | Codecasa *et al*. [13] | 2021 | case report | 2 | 0 | 0 | 2 | 2 | 2 | 2 | 0 | 0 | 10 |
| 7. | Kissami *et al*. [14] | 2021 | case report | 2 | 0 | 0 | 2 | 2 | 2 | 2 | 0 | 0 | 10 |
| 8. | Ishii *et al*. [15] | 2020 | retrospective study | 2 | 2 | 0 | 2 | 2 | 2 | 2 | 0 | 2 | 14 |
| 9. | Aimo *et al*. [14] | 2020 | case series | 2 | 0 | 0 | 2 | 2 | 2 | 2 | 0 | 0 | 10 |
| 10. | Piels *et al*. [16] | 2019 | case report | 2 | 0 | 0 | 2 | 2 | 2 | 2 | 0 | 0 | 10 |
| 11. | Ozdemir *et al*. [17] | 2019 | case report | 2 | 0 | 0 | 2 | 2 | 2 | 2 | 0 | 0 | 10 |
| 12. | Choo *et al*. [18] | 2019 | prospective study | 2 | 2 | 2 | 2 | 2 | 2 | 2 | 0 | 2 | 16 |
| 13. | Li B, *et al*. [19] | 2019 | case report | 2 | 0 | 0 | 2 | 2 | 2 | 2 | 0 | 0 | 10 |
| 14. | Roth *et al*. [20] | 2018 | case report | 2 | 0 | 0 | 2 | 2 | 2 | 2 | 0 | 0 | 10 |
| 15. | Biere *et al*. [21] | 2017 | retrospective study | 2 | 2 | 0 | 2 | 2 | 2 | 2 | 0 | 2 | 14 |
| 16. | Kalvin *et al*. [22] | 2017 | case report | 2 | 0 | 0 | 2 | 2 | 2 | 2 | 0 | 0 | 10 |
| 17. | Munoz *et al*. [23] | 2016 | case report | 2 | 0 | 0 | 2 | 2 | 2 | 2 | 0 | 0 | 10 |
| 18. | Viveiros *et al*. [24] | 2015 | case report | 2 | 0 | 0 | 2 | 2 | 2 | 2 | 0 | 0 | 10 |
| 19. | Akilli *et al*. [25] | 2013 | case report | 2 | 0 | 0 | 2 | 2 | 2 | 2 | 0 | 0 | 10 |
| 20. | Jędrychowska *et al*. [9] | 2021 | retrospective study | 2 | 2 | 0 | 2 | 2 | 2 | 2 | 0 | 2 | 14 |
| 21. | Bossard *et al*. [10] | 2021 | RCT | 2 | 2 | 2 | 2 | 2 | 2 | 2 | 2 | 2 | 18 |
| 22. | Gasior *et al*. [11] | 2020 | retrospective study | 2 | 2 | 0 | 2 | 2 | 2 | 2 | 0 | 2 | 14 |
